# Relationship between initial peritoneal dialysis modality and risk of peritonitis

**DOI:** 10.1038/s41598-020-75918-5

**Published:** 2020-10-30

**Authors:** Maiko Kokubu, Masaru Matsui, Takayuki Uemura, Katsuhiko Morimoto, Masahiro Eriguchi, Kenichi Samejima, Yasuhiro Akai, Kazuhiko Tsuruya

**Affiliations:** 1 Department of Nephrology, Nara Prefecture General Medical Center, 2-897-5 Shichijo-nishimachi, Nara, 630-8581 Japan; 2 Department of Nephrology, Nara Prefecture Seiwa Medical Center, Nara, 636-0802 Japan; 3 Department of Nephrology, Nara Medical University, Kashihara, Nara 634-8521 Japan

**Keywords:** Medical research, Nephrology

## Abstract

Peritonitis is a critical complication of peritoneal dialysis (PD). Investigators have reported the risk of peritonitis in patients on continuous ambulatory peritoneal dialysis (CAPD) versus automated peritoneal dialysis (APD), but the available evidence is predominantly based on observational studies which failed to report on the connection type. Our understanding of the relationship between peritonitis risk and PD modality thus remained insufficient. We studied 285 participants who began PD treatment between 1997 and 2014 at three hospitals in Nara Prefecture in Japan. We matched 106 APD patients with 106 CAPD patients based on their propensity scores. The primary outcome was time to first episode of peritonitis within 3 years after PD commencement. In total, PD peritonitis occurred in 64 patients during the study period. Patients initiated on APD had a lower risk of peritonitis than did those initiated on CAPD in both the unadjusted and adjusted models. The hazard ratio (HR) and 95% confidence interval (CI) for the primary endpoint were 0.30 (0.17–0.53) in the fully adjusted model including connection type. In the matched cohort, APD patients had a significantly lower risk of peritonitis than did CAPD patients (log-rank: *p* < 0.001, HR 0.32, 95% CI 0.16–0.59). The weighting-adjusted analysis of the inverse probability of treatment yielded a similar result (HR 0.35, 95% CI 0.18–0.67). In conclusion, patients initiated on APD at PD commencement had a reduced risk of peritonitis compared with those initiated on CAPD, suggesting APD may be preferable for prevention of peritonitis among PD patients.

## Introduction

Peritonitis is a frequent and serious complication among patients on peritoneal dialysis (PD) and is characterized by fever, severe abdominal pain and cloudy effluents. Some centers report that PD peritonitis accounts for approximately 0.20 episodes per patient-year^1,2^. PD peritonitis is considered a direct or indirect cause of death in 2–16% of patients^3–5^. Furthermore, peritonitis greatly reduces dialytic efficiency via peritoneal fibrosis progression^6,7^ and remains a critical cause of technique failure for some PD patients who must then be promptly switched to hemodialysis. Clinicians must identify risk factors associated with PD-related peritonitis. Peritonitis incidence has been significantly reduced in recent years owing to technological advances in PD connectology, development of new cycler machines and biocompatible PD solutions, and institution of the International Society for Peritoneal Dialysis (ISPD) guidelines for preventing and treating PD-related peritonitis^8^.

Previous reports have investigated the risk of peritonitis in patients on continuous ambulatory peritoneal dialysis (CAPD) versus automated peritoneal dialysis (APD)^9–15^. The available evidence is predominantly based on observational studies^9–13^ but not randomized control studies^14,15^ and is insufficient to evaluate the relationship between peritonitis risk and PD modality. The analysis of these studies is handicapped by failure to report on the connection device in the cyclers used. In addition, to our knowledge, there have been no investigations studying the relationship of CAPD and APD with peritonitis in Japan and with propensity score (PS) method. We therefore examined the association between PD modality at PD commencement and PD-related peritonitis through a multicentered cohort study in Japan with PS matching analysis.

## Results

### Baseline characteristics

**Figure 1:**
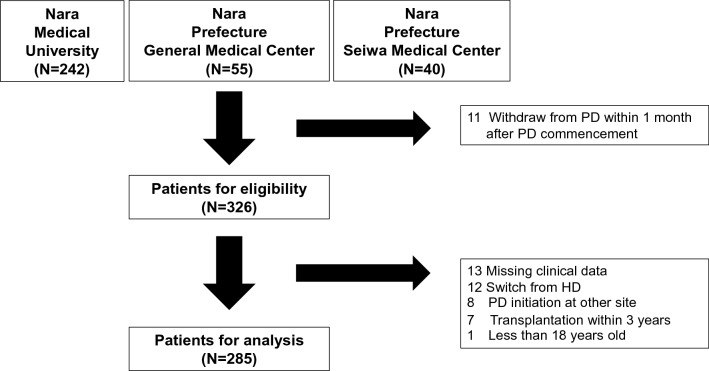
Study Flowchart.

Study flowchart was shown in Fig. 1. Two hundred eight-five PD patients (median age 62 years, interquartile range 60–63 years; 192 men) were analyzed in the present study. Table 1 lists their baseline characteristics at PD commencement. One hundred thirty-three patients were on APD; 152 were on CAPD. Diabetes prevalence and connecting device use were significantly higher in patients on APD than in those on CAPD. In our cohort of 205 patients with available solution data, use of 2.5% dextrose peritoneal dialysis solution bag was similar between APD (4%) and CAPD (8%) patients (*p* = 0.25) but CAPD (27%) patients have significantly high prevalence with use of icodextrin solution compared to APD (6%) patients (*p* < 0.001).

**Table 1:** Baseline characteristics before and after propensity score matching.

	Before PS matching		*p* value	After PS matching		*p* value
	APD	CAPD		APD	CAPD	
Number of patients (%)	133 (47)	152 (53)		106 (50)	106 (50)	
Age, years	63 (54–72)	62 (53–73)	0.85	61 (53–72)	63 (53–73)	0.93
Gender, female, n (%)	40 (30)	56 (37)	0.23	34 (32)	34 (32)	1.00
Diabetes, n (%)	67 (50)	52 (34)	0.006	46 (43)	43 (41)	0.68
Hypertension, n (%)	120 (90)	134 (88)	0.58	95 (90)	95 (90)	1.00
Dyslipidemia, n (%)	45 (34)	47 (31)	0.60	37 (35)	33 (31)	0.56
Overweight, n (%)	34 (26)	36 (25)	0.94	29 (27)	29 (28)	0.97
eGFR, mL/min/1.73 m^2^	4.4 (3.6–5.5)	4.7 (3.7–5.8)	0.18	4.4 (3.8–5.6)	5.6 (3.6–5.6)	0.67
Hemoglobin, g/dL	9.6 (8.4–10.6)	9.7 (8.7–10.7)	0.35	8.4 (7.6–9.7)	8.5 (7.3–9.7)	0.94
Serum albumin, g/dL	3.7 (3.2–4)	3.6 (3.1–4)	0.95	3.7 (3.2–4.0)	3.8 (3.3–4.1)	0.31
C-reactive protein, mg/dL	0.11 (0.02–0.7)	0.2 (0.1–0.7)	0.45	0.1 (0–0.5)	0.12 (0.1–0.6)	0.48
Uric acid, mg/dL	7.8 (6.4–9.3)	7.8 (6.3–9.4)	0.76	8.1 (6.3–9.3)	7.9 (6.3–9.5)	0.90
Calcium, mg/dL	8.7 (7.7–9.2)	8.8 (8.3–9.3)	0.03	8.8 (7.8–9.2)	8.6 (8.0–9.1)	0.88
Phosphorus, mg/dL	5.8 (5.0–7.3)	5.7 (4.6–6.7)	0.07	5.8 (4.9–6.9)	6.0 (4.8–6.9)	0.91
Use of connection device, n (%) (%)	77 (58)	69 (45)	0.03	59 (56)	55 (52)	0.58

Data are shown as the median (interquartile range) or n (%) as appropriate.

PS, propensity score; eGFR: estimated glomerular filtration rate.

The groups did not significantly differ after PS matching (Table 1).

### Outcome and PD modality

**Figure 2:**
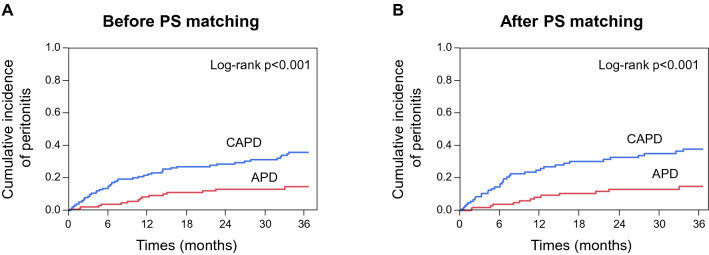
Kaplan–Meier analysis showing the cumulative incidence of the primary endpoint in patients receiving PD according to PD modality before (**A**) and after (**B**) propensity score matching (*p* < 0.001 and *p* < 0.001 by the log-rank test).

During the study period (median 31 months), PD peritonitis occurred in 64 patients including 16 of 133 (0.05 episodes/patient-year) patients on APD and 48 of 152 (0.12 episodes/patient-year) patients on CAPD; using Poisson analysis this difference was significant (*p* = 0.005). In 56 patients with available causative organisms, the number and rate of gram-positive cocci showed a high prevalence in CAPD versus APD patients, but without statistical significance (*p* = 0.32) (Supplementary Figure). Among all patients, Kaplan–Meier analysis demonstrated that APD was significantly associated with a lower probability of peritonitis than was CAPD (log-rank *p* < 0.001; Fig. 2). Both the unadjusted and adjusted models showed that patients who started on APD had a lower risk of peritonitis. The hazard ratio (HR) and 95% confidence interval (CI) for the primary endpoint were 0.35 (0.19–0.61) in the unadjusted model and was 0.33 (0.19–0.59) in model 1 adjusted for age and sex, 0.32 (95% CI 0.18–0.57) in model 2 adjusted for model 1 plus diabetes and overweight and 0.30 (95% CI 0.17–0.53) in model 3 adjusted for model 2 plus use of connection device. The HR adjusted for the model 3 plus center remained significant (HR 0.30 [95% CI 0.17–0.53]). In 205 participants with available solution data the HR was 0.28 (95% CI 0.13–0.61) in the final model plus icodextrin solution.

After PS matching, APD continued to present a significantly lower risk of peritonitis than did CAPD (log-rank *p* < 0.001, HR 0.32, 95% CI 0.16–0.59; Fig. 1). We then used inverse probability of treatment weighting (IPTW) using PS to minimize the differences in patient characteristics. The IPTW-adjusted HR (95% CI) for the primary outcome was 0.35 (0.18–0.67) for APD patients compared with CAPD patients.

**Figure 3:**
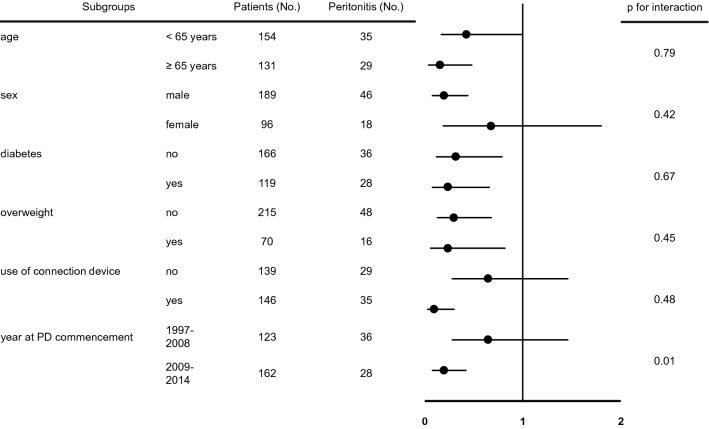
Multivariable-adjusted hazard ratios (95% confidence intervals) of the primary endpoints for APD versus CAPD within subgroups stratified by age, sex, diabetes, overweight patients and connection devise use.

The analyses stratified by age, sex, diabetes, overweight patients, connection device use and year at PD commencement, showed similar associations among participants, excluding female, no use of connection device and earlier years at PD commencement (Fig. 3).

## Discussion

Our study is the first to show that selecting APD rather than CAPD at PD commencement was significantly associated with a lower peritonitis incidence in Japanese PD patients. Adjusting for confounding covariates did not attenuate the HR of APD. Similar results were obtained in the PS-matching and IPTW analyses, suggesting that starting PD patients on APD rather than CAPD can reduce the risk of PD-associated peritonitis within 3 years after PD commencement.

Peritonitis is a major cause of PD failure via structural and functional alterations of the peritoneal membrane^16^, which can lead to life-threatening events^9,17^. Previous reports have shown a relationship between PD modality and peritonitis, but these studies are observational^9–13^ rather than randomized control trials^14,15^. Results of many studies have shown that patients on APD have a lower or similar incidence of peritonitis than do those on CAPD. A randomized control study in the Netherlands revealed that PD peritonitis occurred significantly less often in APD versus CAPD patients (0.51 versus 0.94 episodes per patient-year, respectively)^14^. A retrospective observational study in the United Kingdom^9^ showed that the peritonitis rate was 1:36.7 patient-months for APD-treated patients and 1:28.8 patient-months for CAPD-treated patients, representing an odds ratio of 0.78 favoring APD. Conversely, three recent observational studies found that PD modality was unassociated with a higher likelihood of developing peritonitis^11–13^. Differing study populations, follow-up times, improvements in CAPD and APD connection systems, and advances in nursing care and dialysis treatment may have led to the differing results in these studies. The analysis of these studies fails to report on the connection device in the cycler used. These data should also be interpreted with caution because of differences in the analytical methods. Our multicenter study in Japan showed that patients who started on APD at PD commencement experienced significantly less peritonitis than did those starting on CAPD in both the unmatched and matched patients and subgroup analyses, confirming the robustness of our results.

Two hypotheses may explain why APD was associated with a lower peritonitis incidence than was CAPD. First, the number of connections and disconnections required to perform PD may be the most important determinant of peritonitis rates; APD requires fewer connections and disconnections than does CAPD. Second, APD, especially nocturnal intermittent PD, involves shorter dialysate dwelling times than does CAPD, which requires at least 4 to 5 bag exchanges. Fewer connections and shorter dwelling times may reduce the peritonitis incidence by reducing touch contamination in APD-treated patients. However, we found increased number and rate of peritonitis causing gram-positive cocci in CAPD versus APD patients, but without statistically significant. There may be other mechanisms that contribute to risk reduction of peritonitis in APD.

Several study limitations are noted. First, this was an observational study; therefore, the cause-effect relationship between APD and peritonitis incidence was uncertain. Second, the PD modality was selected by the clinicians and their patients, possibly introducing selection bias into the survey findings, although some clinical data were adjusted. Third, only the PD modality at PD commencement was analyzed; changes in PD modality were not followed. Fourth, the enrollment period was relatively long; thus, advances in dialysis treatment, including the dialysate and devices, might have affected the results. Based on results of the subgroup analysis, APD may contribute to risk reduction of peritonitis in recent years. Fifth, some variables associated with the choice of modality and peritonitis were not evaluated. Socioeconomic data, caregiver status and education level are associated with the choice of modality and incident peritonitis in PD patients^18,19^.

In conclusion, selection of APD rather than CAPD at PD commencement was associated with a lower peritonitis incidence, suggesting that APD may be preferable for prevention of peritonitis in PD patients. Larger prospective randomized studies are needed to ensure the robustness of our results.

## Methods

### Patients

In total, we screened 337 consecutive PD patients who were treated at three centers in Nara Prefecture in Japan between 1 April 1997 and 31 December 2014. Fifty‐two patients were excluded because of the inclusion and exclusion criteria, leaving 285 patients for analysis. The inclusion criterion was that the patients must have received maintenance PD for at least one month. Exclusion criteria were missing clinical data, transition from hemodialysis, PD commencement at other clinics, transplantation within 3 years, and age less than 18 years. Each hospital’s ethics board approved the study (Approval No. 2002–009, No. 316 and No.131).

### Clinical definitions

Baseline demographics and blood sample results were obtained within one month before starting PD through patient interviews and medical records. Hypertension was defined as systolic blood pressure ≥ 140 mmHg, diastolic blood pressure ≥ 90 mmHg, or current treatment with oral antihypertensive drugs. Diabetes was defined as a fasting glucose level ≥ 126 mg/dl or current treatment with oral hypoglycemic medications or insulin. Dyslipidemia was defined as low-density lipoprotein cholesterol ≥ 140 mg/dl or current treatment with lipid-lowering medications.

### Peritoneal dialysis

In the study PD system of two companies including Baxter Healthcare Corporation and Terumo Corporation was used; 253 (89%) patients were on the system of Baxter Healthcare Corporation and the rest were on that of Terumo Corporation. Connection devices were UV Flash (Baxter Healthcare Corporation, Deerfield, Illinois, USA) and the TSCD (Terumo Sterile Connector Device, Terumo Corporation, Tokyo, Japan). The clinicians and patients determined whether to use CAPD or APD with or without a connection device.

### Study outcomes

The primary outcome was time to first episode of peritonitis within 3 years after PD commencement. PD-related peritonitis was defined as an effluent leukocyte count of > 100 cells/mm^3^, with at least 50% being polymorphonuclear leukocytes according to ISPD guidelines^8^. In the entire cohort 2 patients with missing follow-up, 66 deaths and 81 patients who switched hemodialysis were treated as censored cases. All events were confirmed through medical records and self-reporting.

### Statistical analysis

All variables are expressed as medians (interquartile range). Differences between the groups were determined using the Mann–Whitney U test or the χ^2^ test. The rates of peritonitis between groups were compared by using Poisson analysis. Propensity scores were calculated using multivariable logistic regression to estimate probability of receiving APD versus CAPD. Demographics (age and sex), comorbidities (diabetes, hypertension, dyslipidemia and being overweight [defined as having a body mass index of 25 kg/m^2^ or more]), blood parameters (estimated glomerular filtration rate, hemoglobin, serum albumin, C-reactive protein, uric acid, calcium, and phosphorus), and use or no use of a connection device were included as covariates. Covariates were included in the propensity score model. Propensity scores were then used to match APD patients to CAPD patients 1:1 using a greedy nearest-neighbor matching algorithm. Cumulative incidence of the primary endpoint was estimated using the Kaplan–Meier method according to PD modality; differences were assessed using the log-rank test in both the unmatched and matched cohorts. A Cox regression model was used to determine unadjusted and adjusted associations between PD modality and the study endpoint. We initially adjusted for age and sex in model 1. Model 2 consisted of model 1 plus diabetes and overweight and Model 3 consisted of model 2 plus use of connection device. Two-sided *p* values < 0.05 were considered statistically significant. JMP 10.0.02 (SAS Institute, Cary, NC, USA) and IBM SPSS Statistics, version 24 (IBM-SPSS Inc., Armonk, NY, USA) were used to perform all statistical analyses.

### Ethical approval and informed consent

Nara Medical University, Nara Prefecture General Medical Center and Nara Prefecture Seiwa Medical Center Ethics Committee approved the study protocol and waived the requirement for written informed consent as a part of study approval (Approval No. 2002–009, No. 316 and No.131, respectively). All procedures performed in studies involving human participants were in accordance with the ethical standards of each institutional research committee and with 1964 Helsinki declaration and its amendments or comparable ethical standards. Research content has been included on the web page of our department (https://www.nara-hp.jp/about/ethics).

## Supplementary information

Supplementary Information
