# Supplementary material for: Relationship between initial peritoneal dialysis modality and risk of peritonitis
Source: Sci Rep. 2020 Oct 30;10:18763. doi: 10.1038/s41598-020-75918-5 (PMC7599327; doi:10.1038/s41598-020-75918-5)
Supplement: Supplementary file 1 — Supplementary Information [file 41598_2020_75918_MOESM1_ESM.pdf]

Supplementary File

Title: Relationship between initial peritoneal dialysis modality and risk of peritonitis

Authors:

Maiko Kokubu[1], Masaru Matsui[1], Takayuki Uemura[1], Katsuhiko Morimoto[2], Masahiro Eriguchi[3], Kenichi Samejima[3], Yasuhiro Akai[3], Kazuhiko Tsuruya[3]

Institution:

1. Department of Nephrology, Nara Prefecture General Medical Center, Nara, 630-8581, Japan
2. Department of Nephrology, Nara Prefecture Seiwa Medical Center, Nara, 636-0802, Japan,

Department of Nephrology, Nara Medical University, Kashihara, Nara 634-8521, Japan

Supplementary Figure

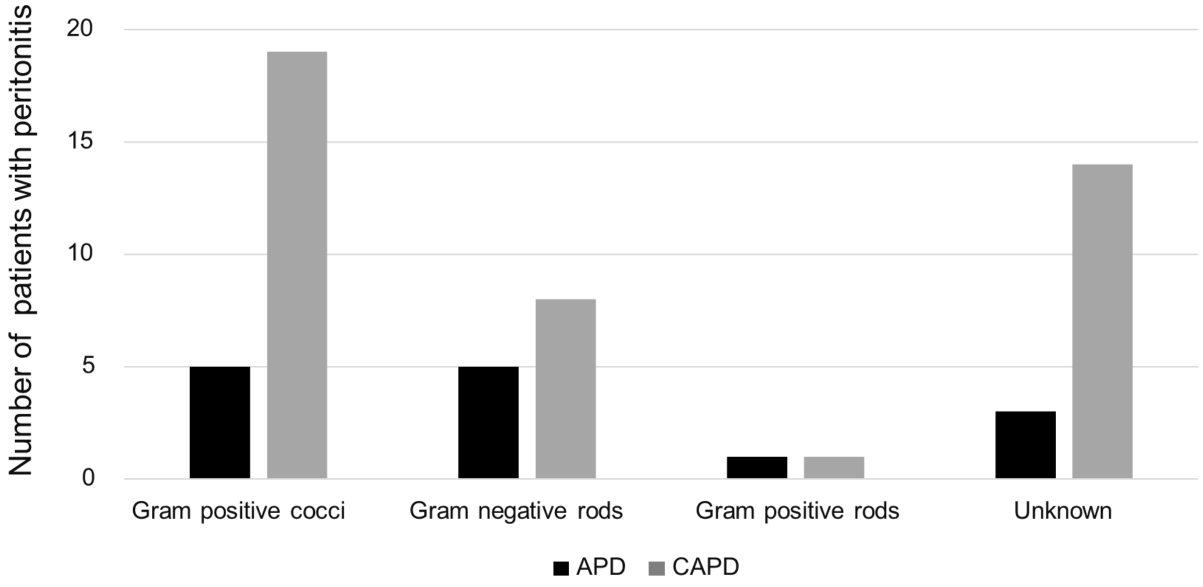

Available causative organisms in PD patients who developed peritonitis during study period.
